# Effect of tolvaptan on renal handling of water and sodium, GFR and central hemodynamics in autosomal dominant polycystic kidney disease during inhibition of the nitric oxide system: a randomized, placebo-controlled, double blind, crossover study

**DOI:** 10.1186/s12882-017-0686-3

**Published:** 2017-08-15

**Authors:** Safa Al Therwani, My Emma Sofie Malmberg, Jeppe Bakkestroem Rosenbaek, Jesper Noergaard Bech, Erling Bjerregaard Pedersen

**Affiliations:** University Clinic in Nephrology and Hypertension, Department of Medical Research, Holstebro Hospital and Aarhus University, Hospital Unit Jutland West, Laegaardvej 12, 7500 Holstebro, Denmark

**Keywords:** Tolvaptan, ADPKD, Nitric oxide, ENaC, AQP2, Blood pressure, Vasoactive hormones

## Abstract

**Background:**

Tolvaptan slows progression of autosomal dominant polycystic kidney disease (ADPKD) by antagonizing the vasopressin-cAMP axis. Nitric oxide (NO) stimulates natriuresis and diuresis, but its role is unknown during tolvaptan treatment in ADPKD.

**Methods:**

Eighteen patients with ADPKD received tolvaptan 60 mg or placebo in a randomized, placebo-controlled, double blind, crossover study. L-NMMA (L-NG-monomethyl-arginine) was given as a bolus followed by continuous infusion during 60 min. We measured: GFR, urine output (UO), free water clearance (C_H2O_), fractional excretion of sodium (FE_Na_), urinary excretion of aquaporin-2 channels (u-AQP2) and epithelial sodium channels (u-ENaCγ), plasma concentrations of vasopressin (p-AVP), renin (PRC), angiotensinII (p-AngII), aldosterone (p-Aldo), and central blood pressure (cBP).

**Results:**

During tolvaptan with NO-inhibition, a more pronounced decrease was measured in UO, C_H2O_ (61% vs 43%) and FE_Na_ (46% vs 41%) after placebo than after tolvaptan; GFR and u-AQP2 decreased to the same extent; p-AVP increased three fold, whereas u-ENaC_γ_, PRC, p-AngII, and p-Aldo remained unchanged. After NO-inhibition, GFR increased after placebo and remained unchanged after tolvaptan (5% vs −6%). Central diastolic BP (CDBP) increased to a higher level after placebo than tolvaptan. Body weight fell during tolvaptan treatment.

**Conclusions:**

During NO inhibition, tolvaptan antagonized both the antidiuretic and the antinatriuretic effect of L-NMMA, partly via an AVP-dependent mechanism. U-AQP2 was not changed by tolvaptan, presumeably due to a counteracting effect of elevated p-AVP. The reduced GFR during tolvaptan most likely is caused by the reduction in extracellular fluid volume and blood pressure.

**Trial registration:**

Clinical Trial no: NCT02527863. Registered 18 February 2015.

## Background

Autosomal dominant polycystic kidney disease (ADPKD) is one of the most frequent hereditary kidney diseases [1, 2]. It is characterized by accelerated cyst growth leading to increased total kidney volume (TKV) and worsening of kidney function [3]. Orthologous ADPKD animal models suggest that vasopressin (AVP) plays a key role in the cytogenesis by promoting cystic epithelial cell proliferation, and by stimulating chloride-driven luminal fluid secretion [4–8]. This response is mediated by the second messenger adenosine 3′,5′- cyclic monophosphate (cAMP) as a consequence of activated vasopressin receptors (V2R). Intracellular aquaporin-2 (AQP2) water channels are in turn translocated to the apical plasma membrane of the collecting duct principal cells [4–7]. Activation of the V2R may also increase the synthesis of nitric oxide (NO), thereby facilitating renal water absorption by membrane insertion of AQP2 [9–14]. NO is synthesized from L-arginine, a NO synthase-catalyzed process competitively inhibited by L-NMMA [13]. Previous clinical studies conducted by our laboratory demonstrated that NO promoted natriuresis and diuresis in healthy subject [15, 16]. Although these results support that NO is involved in the V2R response in the tubular function, it is unknown whether the response is similar in ADPKD patients.

In addition to AQP2, epithelial sodium channels (ENaC) are also present on the apical plasma membrane, but the amount is downregulated in ADPKD patients [17–20].

Tolvaptan, a selective V2 receptor antagonist, has recently been approved by the European Medicines Agency (EMA) for the treatment of ADPKD corresponding to chronic kideney disease stages I-III [21]. Tolvaptan reduced cyst growth corresponding to TKV and the decline in GFR by antagonizing the AVP-induced renal cAMP accumulation [3]. The activity in AQP2 water channels and epithelial sodium channels has not previously been investigated in ADPKD patients before and after systemic NO inhibition during tolvaptan treatment. We hypothesized that 1. The function of AQP2- and ENaC channels is abnormal in ADPKD patients; 2. Tolvaptan decreases renal water- and sodium absorption; 3. This response is reduced by systemic NO-inhibition; and 4. Tolvaptan’s effect on renal water and sodium absorption is partially counteracted by increased blood pressure (BP) and vasoactive hormones at baseline and after systemic NO inhibition.

In the present randomized, placebo-controlled, double-blinded, crossover study of ADPKD patients, the aim was to measure tolvaptan’s effect at baseline and during NO inhibition on 1. GFR and tubular handling of water and sodium (GFR (^51^Cr-EDTA-clearance), urinary output (UO), free water clearance (C_H2O_), fractional excretion of sodium (FENa), urinary excretion of protein fragments from aquaporin 2 (u-AQP2) and epithelial sodium channels (u-ENaCγ), and urinary nitrate (u-nitrate)); 2. Blood pressure (brachial blood pressure (bBP), central BP (cBP), pulse wave velocity (PW), augmentation index (AI)); and 3. Vasoactive hormones in plasma (vasopressin (p-AVP), renin (PRC), angiotensinII (p-AngII), and aldosterone (p-Aldo)).

## Methods

### Subjects

#### Inclusion criteria

ADPKD patients meeting the following inclusion criteria were included:

1. Caucasian men and women
2. Age 18–65 yrs.
3. BMI 18.5–35.5 kg/m^2^
4. ADPKD diagnosed by genetic testing for PKD1 (> 85%) and PKD2 mutations, or presence of one of the following ultrasonographic findings in accordance to the classical Ravine criteria [22]: a) patients with a negative family history of ADPKD with more than 10 cysts in each kidney, and exclusion of other causes of extra-renal or renal cyst formations, b) patients with a family history of ADPKD: 15–39 yrs. and 3 cysts or more unilaterally or bilaterally/ 40–59 yrs. and 2 or more cysts in each kidney/ 60 yrs. and at least 4 cysts in each kidney.
5. Kidney function corresponding to CKD stages 1–3 (eGFR > 30 mL/min/1,73 m2).

#### Exclusion criteria

Exclusion criteria were: 1) clinical signs of diseases in the heart, lungs, endocrine organs, brain or neoplastic disease; 2) clinically significant abnormalities in blood or urine sample at the inclusion; 3) previous cerebrovascular insults; 4) previous clinical evidence of aneurysm; 5) alcohol or drug abuse; 6) smoking; 7) pregnancy or breastfeeding; 8) clinically significant changes in the electrocardiogram; 9) medication except antihypertensive agents and oral contraceptives; and 10) blood pressure > 170/105 mmHg despite treatment with metoprolol and/or amlodipine.

### Study design

A randomized, double-blinded, placebo-controlled, crossover study was performed in patients with ADPKD before and after NO inhibition using L-NMMA. To test the difference between tolvaptan 60 mg and placebo, each patient participated in two examination days with an intermediate wash-out period of at least 3 weeks to eliminate any carryover effects.

### Medications

Tolvaptan (SAMSCA®, Otsuka, Tokyo, Japan) 60 mg and placebo were coated in identical gelatine capsules and were orally administered at 8:00 AM. L-NMMA (Bachem, Weil am Rhein, Germany) was dissolved in isotonic saline solution and given intravenously at 11:00 AM.

### Number of subjects

C_H2O_ was used as the main effect variable. With a minimal relevant difference of 6 ml/min with an estimated standard deviation (SD) of 4 ml/min, and using a level of significance of 5% and a statistical power of 90%, 12 subjects were needed. To allow for possible drop-outs or incomplete voiding during the examination days, 18 subjects were included.

### Recruitment

Eligible ADPKD patients were recruited from the Outpatient Nephrology Clinic of the Department of Medicine at Holstebro Hospital, Denmark.

### Effect variables

The primary effect variable was C_H2O._ The secondary effect variables were 1) renal function (^51^Cr-EDTA clearance, UO, u-AQP2, u-ENaC_γ_, FE_Na_, u-nitrate), 2) hemodynamics (bBP, cBP, PWV, AI, and 3) vasoactive hormones (PRC, p-ANG II, p-Aldo, p- AVP).

### Antihypertensive medications

Antihypertensive medications including diuretics, angiotensin-converting enzyme (ACE) inhibitors, and angiotensin-II inhibitors were discontinued or substituted with metoprolol 50 mg and/or amlodipine 5 mg 14 days prior to each examination day. During the study period, bBP was examined weekly. In addition, 24-h blood pressure was performed 1 week after discontinuation or substitution of the usual antihypertensive treatment prior to the first examination day. Blood pressure of >170/105 mmHg, metoprolol 50 mg, and/or amlodipine 5 mg was given and increased up to metoprolol 150 mg and/or amlodipine 10 mg. Continued blood pressure > 170/105 mmHg despite treatment with metoprolol 150 mg and/or amlodipine 10 mg led to withdrawal from the study. The usual antihypertensive treatment was resumed immediately after the end of the examination day. Patients were given the same dose of metoprolol and/or amlodipine 14 days prior to the second examination day.

### Diet

Prior to each examination day, ADPKD patients consumed a 4-day standardized diet of 11,000 KJ day^−1^. The diet was delivered from our facilities, and in conformity with general dietary guidelines, it was composed of 15% proteins, 55% carbohydrates and 30% fat. The sodium content was 150 mmol day^−1^. No additional sodium or other spices were allowed. The daily fluid intake was also standardized to 2.5 L, including a maximum of two cups of coffee or tea. No alcohol or soft drinks were allowed during the diet period.

### Experimental procedure

The procedures were identical on the two examination days. A 24-h urine sample was collected and a fasting period of 8 h was allowed prior to each examination day. The two examinations were conducted at our facility from 7:45 AM to 1:00 PM.

At 8:00 AM, ADPKD patients were given placebo or tolvaptan 60 mg. An intravenous catheter was placed in each arm to collect blood samples and infuse ^51^Cr-EDTA. An oral water load of 175 ml was given at 8:00 AM and every 30 min.

BP was measured every 30 min from 8:30 AM to 1:00 PM. At 11:00 AM, a bolus infusion of L-NMMA 4.5 mg/kg was given, followed by continuous infusion (3 mg/kg/h) during 1 h. The dose was based on results from a dose-finding study of healthy subjects conducted by our laboratory [23]. BP was measured every 5 min during infusion of L-NMMA, and every 15 min after infusion of L-NMMA.

From 8:30 AM to 1:00 PM, blood samples were drawn every 30 min and were analyzed for p-Na, p-osm, p-^51^Cr-EDTA, p-creatinine, and p-albumin. Every 60 min; at 11:00 AM (baseline), at 12:00 AM (after end of L-NMMA infusion), and at 1:00 PM (60 min after end of L-NMMA infusion), blood samples were collected to measure PRC, p-Aldo, p-Ang II, and p-AVP.

From 9:30 AM to 1:00 PM, urine samples were collected by voiding in standing or sitting position every 30 min after collecting BP measurements and blood samples. Otherwise subjects were kept in supine position in a quiet and temperature-controlled room (22–25 °C). Baseline periods were means of the first three clearance periods. The urine samples were analyzed for sodium, osmolality, AQP2, ENaC_γ_, creatinine, ^51^Cr-EDTA, and u-nitrate.

Applanation tonometry with SpygmoCor® (CPV System®, AtCor Medical, Sydney, Australia) was performed at 10:10 AM and at 11: 40 AM to measure cBP, PVW, and AI.

### Measurements

#### Renal function

^51^Cr-EDTA clearance was measured using the constant infusion clearance technique with 51Cr-EDTA as a reference substance.

C_H2O_ was determined using the formula C_H2O_ = UO- C_osm_, where C_osm_ is the osmolar clearance.

Clearance (C) of substance X was calculated as C_X_ = U_X_/ (P_X_ x UO), where U_X_ denotes concentration of x in urine, P_X_ denotes concentration of x in plasma, and UO is urine excretion rate.

Fractional excretion of sodium was determined according to the following formula: FE_Na_ = C_Na_/ ^51^Cr-EDTA-clearance ×100%, where C_Na_ is sodium clearance.

#### Urinary excretion of AQP2

Urine samples were kept frozen at −20 °C until assayed. U-AQP2 were measured by RIA as previously described [24, 25]. The AQP2 antibody was a gift from Professor Soren Nielsen and Professor Robert Fenton, The Water and Salt Center, Aarhus University. It was raised in rabbits to synthetic peptide corresponding to the 15 COOH-terminal amino acids in human AQP2 to which was added an NH2- terminal cysteine for conjugation and affinity purification. Minimal detection level was 34 pg/tube. The coefficients of variation were 11.7% (inter-assay) and 5.9% (intra-assay).

#### Urinary excretion of ENaCγ

U-ENaCγ was measured by a modification of the RIA described previously [26, 27]. ENaCγ was synthesized and purchased by Lofstrand, Gaithersburg, Maryland, USA. The ENaCγ antibody was a gift from Professor Soren Nielsen and Professor Robert Fenton, The Water and Salt Center, Aarhus University. It was raised against a synthetic peptide in rabbits, and the affinity purified as described previously [28]. Iodination of ENaCγ was performed by the chloramine T method using 40 μg of ENaCγ and 37 MBq ^125^I. The reaction was stopped by addition of 20% human serum albumin. 125I–labeled ENaCγ was separated from the iodination mixture by the use of a Sephadex G-25 Fine column. The assay buffer contained 40 mM sodium phosphate (pH = 7.4), 0.2% human albumin, 0.1% Triton X-100, and 0.4% EDTA. A 1.5% solution of bovine gamma globulin (Sigma) and 25% polyethylene glycol 6000 (Merck) also containing 0.625% Tween 20 (Merck) was prepared using the 40 mM phosphate buffer. Urine samples were kept frozen at −20 °C. After thawing out, samples were centrifuged for 10 min at 1.6 × 100 g (3000 rpm). Depending on osmolality of the supernatant, a sample volume was freeze-dried and kept at −20 °C until assayed. The mixture of 300 μl of freeze-dried or standard urine elutes dissolved in 300 μl assay buffer and 50 μl of antibody was incubated for 24 h at 4 °C. Thereafter, 50 μl of the tracer was added, and the mixture was incubated at 4 °C for a further 24 h. Bovine gamma globulin (100 μl) and 2 ml polyethylene glycol 6000 was added. After 30 min, the mixture was centrifuged at 4100 g for 20 min at 4 °C. The precipitate (bound fraction) was counted in a gamma counter after the supernatant (free fraction) was poured off. The unknown content in urine extracts was read from a standard curve. For 6 consecutive standard curves, the zero standard was 49.9 ± 1.6%. For increasing amounts of the ENaCγ standard, the binding inhibition was: 47.5 ± 1.3% (31 pg/tube), 44.0 ± 1.7% (62 pg/tube), 40.6 ± 1.4% (125 pg/tube), 34.6 ± 1.3% (250 pg/tube), 27.0 ± 1.5% (500 pg/tube), 20.4 ± 1.0% (1000 pg/tube), 13.6 ± 0.7% (2000 pg/tube), 8.4 ± 0.5% (4000 pg/tube), and 5.2 ± 0.5% (8000 pg/tube). The ID 50, i.e. the concentration of standard needed for 50% binding inhibition was 626 ± 32 pg/tube (*n* = 6). The nonspecific binding determined by performing the RIA without antibody was 1.0 ± 0.6% (*n* = 6). The inter-assay variation was determined by quality controls from the same urine pool spiked with ENaCγ standard. In consecutive assays, the coefficient of variation was as follows: 10% (6 assays) at a mean level of 338 pg/tube, 9% (6 assays) at a mean level of 743 pg/tube. The intra-assay variation was determined using samples from the same urine pool in several assays at different concentration levels. CV was 11% at a mean level of 110 pg/tube (*n* = 10). CV was 8.3% at a mean level of 118 pg/tube (*n* = 10). CV was 5.2%at a mean level of 325 pg/tube (*n* = 10). CV was 5.5% at a mean level of 754 pg/tube (*n* = 10). CV was 3.4% at a mean level of 942 pg/tube (*n* = 10). In addition, coefficients of variation were calculated based on duplicate determinations in different assays to 5.0% (*n* = 6) in the range 125–135 pg/tube, and 5.6% (*n* = 6) in the range 290–380 pg/ tube. The sensitivity calculated as the smallest detectable difference at the 95% confidence limit was 24 pg/tube in the range 90–130 pg/tube (*n* = 10), 33 pg/tube in the range 300–350 pg/tube (*n* = 10), 65 pg/tube in the range 890–1000 pg/tube (*n* = 10), The lower detectable limit of the assay was 35 pg/tube. It was calculated using the average zero binding for 6 consecutive assays minus 2 SD. The volume of urine used from the same pool varied with 15 different volumes in the range 250–5000 μl, and the mean concentration measured was 151 ± 18 pg/ml. There was a highly significant correlation between the extracted volume of urine and the amount of pg/tube (*r* = 0.986, *n* = 15, *p* < 0.000). When ENaCγ in the range 62.5–1000 pg was added to urine, a highly significant correlation was found between the measured and the expected values (*r* = 0.903, *n* = 20, *p* < 0.000).

#### Vasoactive hormones in plasma

Blood samples collected to measure vasoactive hormones were centrifuged for 10 min at 2200 G and 4 °C. Plasma was separated from blood cells and kept frozen until assayed. PRC was determined with an immunoradiometric assay from CIS Bio International, Gif-Sur-Yvette Cedex, France. The minimal detection level was 1 pg/tube. The coefficients of variation were 0.9–3.6% for the intra-assay and 3.7–5.0% for the inter-assay in the range of 4–236 pg/ml. Aldo was determined by RIA using a kit from Demeditec Diagnostics GmbH, Kiel, Germany. The minimal detection level was 25 pmol/L. The coefficients of variation were 9.0% for the inter-assay and 8.5% for the intra-assay. Ang II and AVP were extracted from plasma and subsequently determined by radioimmunoassay as previously described [29, 30]. Ang II was obtained from the Department of Clinical Physiology, Glostrup Hospital, Denmark. The minimal detection level was 2 pmol/ L. The coefficients of variation were 12% for the inter-assay and 8% for the intra-assay. The antibody against AVP was a gift from Jacques Dürr, Miami, Fl, USA. The minimal detection level was 0.2 pmol/L. The coefficients of variation were 13% for the inter-assay and 9% for the intra-assay.

#### Other biochemical measurements

Plasma osmolality was determined by using freeze-point depression (Multi-Sample Osmometer, Model 3900, Advanced Instruments, MA, USA). Urinary excretion rate of nitrate was determined by using a colorimetric assay using a kit from R&D Systems (Minneapolis, MN, USA) as previously decribed [31]. Sodium, albumin, hemoglobin, and glucose were measured by routine methods in Department of Clinical Biochemistry, Holstebro Hospital, Denmark.

#### Brachial and central blood pressure

Brachial BP was measured using an oscillometer (Omron 705IT) and recorded in accordance with recommendations from the European Society of Hypertension. Brachial systolic and diastolic blood pressures were recorded as the average of duplicate measures. Central BP was measured using applanation tonometry. Recordings of carotid-femoral PWV and PWA were obtained by applanation tonometry (SphygmoCor® CPV System®, AtCor Medical, Sydney, Australia) as previously described [15].

#### Statistics

Statistical analyses were performed using IBM SPSS statistics version 23.0.0 (SPSS Inc., Chicago, IL, USA). General Linear Model Repeated Measures were used for comparison between and within subjects to test differences between placebo and tolvaptan treatment at baseline and during and after L-NMMA infusion. Paired sample t-test or Wilcoxon’s signed rank test was performed to compare tolvaptan and placebo treatment at baseline and during and after L-NMMA infusion. Post-hoc Bonferoni test was performed to compare differences between baseline versus during and after L-NMMA infusion. Statistical significance was at <0.05 in all analyses. Data with normal distribution are presented as means ± SD/SEM or medians with 25th and 75th percentiles. Non-parametric test was performed for data with non-normal distribution, and such data are reported as medians with 25th and 75th percentiles.

## Results

### Demographics

Twenty-one ADPKD patients with CDK stage I-III were included in the study. Three of the patients were excluded due to withdrawal of consent. Eighteen patients completed the study; 6 males and 12 females. Their mean age was 47 years (range 21–62 years), weight 85.2 ± 12.7 kg, BMI 28 ± 5 kgm^2^, p-sodium 141 ± 1.7 mmol/L, p-potassium 3.8 ± 0.2 mmol/L, p-creatinine 84.2 ± 23.8 μmol/L, systolic brachial blood pressure (SBP) 137 ± 13 mmHg, diastolic brachial blood pressure (DBP) 88 ± 7 mmHg.

### Urine collection before the examination day

24-h urine samples were collected prior to each examination day. Values are shown in Table 1. No differences were measured in UO, C_H2O_, u-AQP2, u-Na, and u-K between the treatment periods.

**Table 1:** Urine output, free water clearance (C_H2O_), urinary AQP2 excretion per minute (u-AQP2), urinary sodium excretion (u-Na), and urinary potassium excretion (u-K) during 24-h urine collection in a randomised, placebo-controlled, double-blind, crossover study of 18 ADPKD patients

	Before each examination day		p (paired t-test)
	Placebo	Tolvaptan 60 mg	
Urine Output (ml/24 h)	2504 ± 518	2446 ± 615	0.57
C_H2O_ (ml/min)	−0.22 ± 0.55	−0.19 ± 0.57	0.72
u-AQP2 (ng/min)	1.10 ± 0.26	1.09 ± 0.28	0.88
u-Na (mmol/24 h)	117 ± 34	111 ± 28	0.46
u-K (mmol/24 h)	66 ± 17	65 ± 18	0.77

Values are means with ± SD. Paired t-test was used for comparison between groups

### Body weight during the examination day

No difference was measured in body weight at the beginning and at the end of the examination day after placebo (83.2 ± 12,8 kg vs 83.4 ± 12.8 kg). After tolvaptan 60 mg, body weight decreased significantly at the end of the examination day compared with the beginning of the examination day (83.1 ± 12.1 kg vs 82.1 ± 12.1 kg, *p* = 0.001).

### ^51^Cr-EDTA clearance

**Fig. 1:**
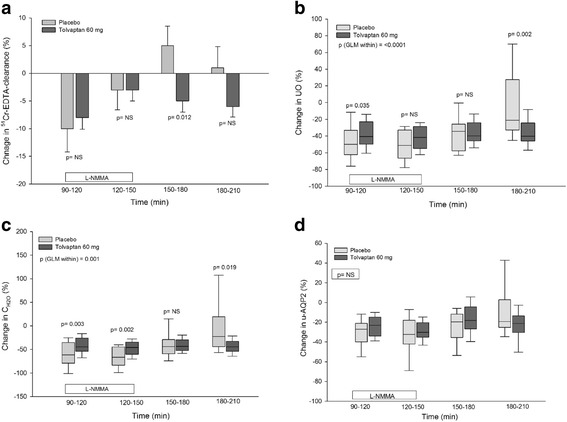
Effect of tolvaptan 60 mg during and after NO inhibition on GFR (^51^ Cr-EDTA-clearance) (**a**), UO (**b**), C_H2O_ (**c**) and u-AQP2 (**d**) in ADPKD. Data are given as mean ± SEM or medians with 25th and 75th percentiles. General linear model (GLM) with repeated measures was performed for comparison within and between groups. Paired t-test was used for comparison between tolvaptan and placebo treatment during L-NMMA infusion period (90–150 min) and post infusion period (150–210 min)

**During baseline,** ^51^Cr-EDTA clearance was the same after placebo and tolvaptan 60 mg (Table 2). **During L-NMMA infusion,** ^51^Cr-EDTA clearance decreased significantly and similarily after both treatments during the first 30 min of L-NMMA infusion (paired t-test: placebo *p* = 0.028 and tolvaptan *p* = 0.001). There was no difference in the relative changes in ^51^Cr-EDTA clearance between groups (Fig. 1a).

**Table 2:** Effect of tolvaptan 60 mg at baseline, during, and after systemic inhibition of NO synthesis on GFR (^51^ CrEDTA-clearance), urinary output (UO), free water clearance (C_H2O_), urinary aquaporin-2 excretion rate (u-AQP2), fractional excretion of sodium (FE_Na_), and urinary ENaCγ excretion rate (u-ENaCγ) in a randomized, placebo-controlled, double-blind, crossover study of 18 ADPKD patients

Periods	Baseline	L-NMMA		Post infusion		*p* (GLM-within)
	0–90 min	90–120 min	120–150 min	150–180 min	180–210 min	
^51^Cr-EDTA-clearance (ml/min/ 1.73 m^2^)						
Placebo	73 ± 20	66 ± 22	72 ± 24	76 ± 17	73 ± 19	0.154
Tolvaptan 60 mg	72 ± 19	67 ± 19	70 ± 19	68 ± 19	67 ± 19	
p (GLM between)	0.684					
p (paired t-test, between)	0.740	0.758	0.643	0.005	0.016	
UO (ml/min)						
Placebo	5.6 ± 1.4	2.9 ± 1.2***	2.8 ± 1.2***	3.6 ± 1.4***	5.1 ± 1.7	< 0.0001
Tolvaptan 60 mg	11.1 ± 1.8	7.0 ± 2.2***	6.3 ± 1.9***	7.1 ± 1.7***	7.0 ± 2.0***	
p (GLM between)	< 0.0001					
p (paired t-test, between)	< 0.0001	< 0.0001	< 0.0001	< 0.0001	0.009	
C_H2O_ (ml/min)						
Placebo	3.0 ± 1.2	1.2 ± 0.8***	1.1 ± 0.7***	1.8 ± 0.9*	2.9 ± 1.4	< 0.0001
Tolvaptan 60 mg	8.4 ± 1.7	4.8 ± 1.6***	4.3 ± 1.4***	4.8 ± 1.0***	4.7 ± 1.2***	
p (GLM between)	< 0.0001					
p (paired t-test, between)	< 0.0001	< 0.0001	< 0.0001	< 0.0001	0.012	
u-AQP2 (ng/min)						
Placebo	1.28 ± 0.37	0.87 ± 0.25***	0.85 ± 0.28***	0.94 ± 0.35*	1.12 ± 0.40	< 0.0001
Tolavptan 60 mg	1.15 ± 0.32	0.87 ± 0.23*	0.82 ± 0.22***	0.93 ± 0.23*	0.89 ± 0.26*	
p (GLM between)	< 0.0001					
p (paired t-test, between)	0.075	0.931	0.702	0.887	0.015	
FE_Na_ (%)						
Placebo	1.39 (1.18; 2.33)	0.91* (0.84; 1.50)	0.78* (0.56; 0.97)	0.51*** (0.28; 0.78)	1.10 (0.88; 1.50)	
Tolvaptan 60 mg	1.18 (0.83; 1.6)	0.86 (0.69; 1.11)	0.75 (0.45; 1.12)	0.44* (0.30; 0.81)	1.21 (0.81; 1.74)	
p (Wilcoxon’s signed rank test, between)	0.122	0.948	0.777	0.948	0.267	
ENaC_γ_ (ng/min)						
Placebo	0.78 (0.67; 0.79)	0.61 (0.45; 0.70)	0.60 (0.43; 0.76)	0.65 (0.37; 0.70)	0.73 (0.63; 0.91)	
Tolvaptan 60 mg	0.75 (0.65; 1.0)	0.59 (0.52; 0.93)	0.66 (0.51; 0.81)	0.68 (0.56; 0.77)	0.69 (0.59; 0.86)	
p (Wilcoxon’s signed rank test, between)	0.711	0.112	0.306	0.248	0.744	

Data are given as mean ± SD or median with 25th and 75th percentiles in parentheses. General linear model (GLM) with repeated measures was performed for comparison within and between groups. Post-hoc Bonferoni test (*) was used for comparison of infusion period (90–150 min) vs baseline period (0–90 min) and post infusion period (150–210 min) vs baseline period

Paired t-test or Wilcoxon’s signed rank test was performed for comparison between tolvaptan and placebo treatment at baseline period (0–90 min), L-NMMA infusion period (90–150 min) and post infusion period (150–210 min)

*p<; 0.05; ***p < 0.0001

**During the post infusion period,** ^51^Cr-EDTA clearance was unchanged after placebo and decreased after tolvaptan during the entire post infusion period (paired t-test: during the post infusion period 150–180 min *p* = 0.008 and during the post infusion period 180–210 min *p* = 0.001). The relative changes in ^51^Cr-EDTA clearance were only differed during the first 30 min of the post-infusion period (*p* = 0.012).

### Tubular water excretion

Absolute and relative values of UO and C_H2O_ are presented in Tables 2 and Fig. 1b and c.

**During baseline,** UO and C_H2O_ were significantly lower after placebo than after tolvaptan treatment. **During L-NMMA infusion 90–120 min,** UO and C_H2O_ decreased after both treatments. The relative decrease in UO and C_H2O_ from baseline to NO inhibition was significantly higher after placebo than after tolvaptan (*p* = 0.035 and 0.003). However, C_H2O_ also decreased relatively more during the last 30 min of the L-NMMA infusion period (120–150 min), unlike UO.

**During the post infusion period 180–210 min,** UO and C_H2O_ increased towards baseline level after placebo, but remained suppressed after tolvaptan at the level that was measured during NO inhibition.

### Tubular sodium excretion

**Fig. 2:**
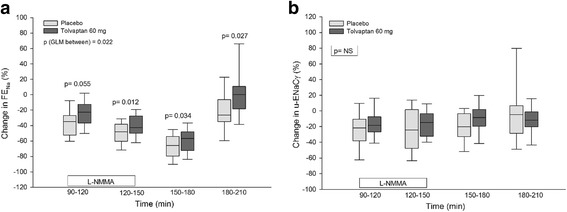
Effect of tolvaptan 60 mg during and after NO inhibition on FE_Na_ (**a**) and u-ENaCγ (**b**) in ADPKD. Data are given as medians with 25th and 75th percentiles. General linear model (GLM) with repeated measures was performed for comparison within and between groups. Paired t-test was used for comparison between tolvaptan and placebo treatment during L-NMMA infusion period (90–150 min) and post infusion period (150–210 min)

Absolute and relative values of FE_Na_ are presented in Table 2 and Fig. 2a.

**During baseline,** no difference was measured in FE_Na_ during placebo and tolvaptan treatment.

**During L-NMMA infusion 120 min,** FE_Na_ significantly decreased after placebo, but remained unchanged after tolvaptan (placebo: 0.48% (corresponding to a percentage decrease of 35%) compared with tolvaptan: 0.32% (26%)). The relative changes from baseline to NO inhibition showed a tendency to lower level of FE_Na_ after placebo (*p* = 0.055). During the remaining L-NMMA infusion period 180–210 min, FE_Na_ decreased significantly more after placebo than after tolvaptan.

**During the post-infusion period,** FE_Na_ remained relatively lower after placebo. During the last 30 min of the post-infusion period, FE_Na_ increased to baseline level after tolvaptan and remained 26% lower compared with baseline level after placebo.

### U-AQP2 and u-ENaCγ

Absolute and relative values of u-AQP2 and u-ENaCγ are presented in Table 2 and Figs. 1d and 2b.

**During baseline**, we saw a tendency towards a higher level of u-AQP2 after placebo treatment (*p* = 0.075). **During L-NMMA infusion**, u-AQP2 decreased to the same extent in both absolute and relative values. **During the post-infusion period (180–210 min),** u-AQP2 returned to baseline level after placebo, but remained lower after tolvaptan treatment (*p* = 0.015). However, no difference was measured in the relative values between treatments, despite a considerable difference in u-AQP2 which may be due to a large variation in u-AQP2.

**During baseline**, u- ENaCγ was alike in the two treatment groups (0.78 ng/min vs 0.75 ng/min). **During L-NMMA infusion,** no difference in either absolute or relative measures existed between treatments. The same result was measured in the post-infusion period.

### Plasma sodium and plasma osmolarity

**During baseline,** p-sodium and p-osm were significantly lower after placebo than after tolvaptan (Table 3). **During L-NMMA infusion,** p-sodium and p-osm remained lower after placebo and this response was sustained throughout the examination day.

**Table 3:** Effect of tolvaptan 60 mg at baseline, during, and after systemic inhibition of NO synthesis on plasma concentration of sodium and plasma osmolality in a randomized, placebo-controlled, double-blind, crossover study of 18 ADPKD patients

Periods	Baseline	L-NMMA		Post infusion		p (GLM-within)
	0–90 min	90–120 min	120–150 min	150–180 min	180–210 min	
p-sodium (mmol/l)						
Placebo	139 ± 2	138 ± 2	138 ± 2	138 ± 1	137 ± 2	< 0.0001
Tolvaptan 60 mg	141 ± 2	141 ± 2	141 ± 2	142 ± 2	141 ± 2	
p (GLM between)	< 0.0001					
p (aired t-test, between)	0.001	< 0.0001	< 0.0001	< 0.0001	< 0.0001	
p- osm (mosm/kg)						
Placebo	285 ± 5	283 ± 4	282 ± 5	283 ± 4	281 ± 4	< 0.0001
Tolvaptan 60 mg	288 ± 5	290 ± 5	289 ± 5	290 ± 5	289 ± 5	
p (GLM between)	< 0.0001					
p (paired t-test, between)	0.024	< 0.0001	< 0.0001	< 0.0001	< 0.0001	

Data are given as mean ± SD. General linear model (GLM) with repeated measurements was used for comparison within and between groups. Post-hoc Bonferoni test was used for comparison of infusion period (90–150 min) vs baseline period (0–90 min) and post infusion period (150–210 min) vs baseline period

Paired t-test was performed for comparison between treatments at baseline period (0–90 min), L-NMMA period (90–150 min) and post-infusion period (150–210 min)

### Urinary excretion rate of nitrate

**During baseline**, a significantly higher urinary excretion rate of NO_3_ (u-NO_3_) was measured after placebo treatment (placebo 0.61 ± 0.23 μmol/min vs tolvaptan: 0.50 ± 0.14 μmol/min, *p* = 0.027).

**During L-NMMA infusion**, u-NO_3_ decreased significantly from the baseline level after both treatments (placebo: 0.39 ± 0.16 μmol/min and tolvaptan: 0.35 ± 0.11 μmol/min, *p* = 0.001). However, the decrease in u-NO_3_ was identical (*p* = 0.185). The same response was measured during the post-infusion period (placebo: 0.36 ± 0.15 μmol/min and tolvaptan: 0.35 ± 0.10 μmol/min).

### Vasoactive hormones

**Fig. 3:**
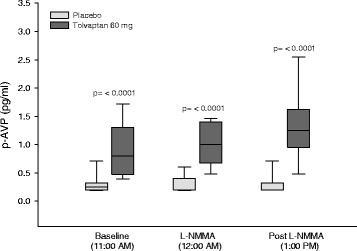
Effect of tolvaptan 60 mg on p-AVP at baseline, during and after NO inhibition in ADPKD. Data are given as median with 25th and 75th percentiles. Wilcoxon’s signed rank test used for comparison between treatment groups prior to L-NMMA infusion, at the end of L-NMMA infusion and 1 h after the end of L-NMMA infusion

**During baseline,** PRC, p-Ang II and p-Aldo were the same (Table 4). However, p-AVP was approximately 3-fold higher after tolvaptan and the difference was statistically highly significant (Fig. 3).

**Table 4:** Effect of tolvaptan 60 mg at baseline, during and after systemic inhibition of NO synthesis on plasma concentrations of renin (PRC), angiotensin II (P-AngII) and aldosterone (P-Aldo) in a randomized, placebo-controlled, double-blind, crossover study of 18 ADPKD patients

Periods	Prior to L-NMMA infusion period	At the end of L-NMMA infusion period	1 h after L-NMMA infusion period	P (GLM-within)
PRC(pg/ml)				
Placebo	8.8 ± 5.3	7.4 ± 4.0	7.7 ± 4.3	0.610
Tolvaptan 60 mg	10.1 ± 5.8	8.9 ± 6.1	8.4 ± 4.9	
p (GLM between)	0.489			
p (paired t-test, between)	0.481	0.312	0.523	
P-AngII (pg/ml)				
Placebo	7.8 ± 3.8	7.4 ± 3.8	7.1 ± 3.1	0.801
Tolvaptan 60 mg	9.1 ± 5.9	8.9 ± 4.7	8.6 ± 4.3	
p (GLM between)	0.327			
p (paired t-test, between)	0.377	0.151	0.261	
P- Aldo (pmol/L)				
Placebo	125 (68; 168)	127 (99; 224)	119 (87; 224)	
Tolvaptan 60 mg	115 (87; 180)	147 (115; 205)	178 (108; 264)	
p (Wilcoxon’s signed rank test, between)	0.420	0.433	0.170	

Data are given as mean ± SD or median with 25th and 75th percentiles in parentheses. General linear model (GLM) with repeated measures was performed for comparison within and between groups. Post-hoc Bonferoni test was used for comparison between L-NMMA infusion period (at the end of L-NMMA infusion period) vs baseline (prior to L-NMMA infusion period) and at baseline vs post infusion period (1 h after L-NMMA infusion period) vs baseline period, none of the *p*- values were significant. Paired t-test was performed to test differences between treatment groups

**During L-NMMA infusion**, the response measured during baseline conditions was sustained throughout the NO-inhibition period and also in the post-infusion period.

### Brachial and central blood pressure

**During baseline**, no significant difference was measured in SPB, DBP, and pulse rate (Table 5). The values were unaffected both during L-NMMA infusion, and the post-infusion period.

**Table 5:** Effect of tolvaptan 60 mg at baseline, during, and after systemic inhibition of NO synthesis on brachial systolic blood pressure (SBP), diastolic blood pressure (DBP), and pulse rate in a randomized, placebo-controlled, double-blind, crossover study of 18 ADPKD patients

Periods	Baseline	L-NMMA		Post infusion		p (GLM-within)
	Prior to L-NMMA infusion	At the beginning of infusion	At the end of infusion	30 min after the end of L-NMMA infusion	60 min after the end of L-NMMA infusion	
SBP (mmHG)						
Placebo	137 ± 12	146 ± 17	149 ± 16	147 ± 16	146 ± 13	0.407
Tolvaptan 60 mg	135 ± 9	144 ± 15	144 ± 14	142 ± 15	144 ± 13	
p (GLM between)	0.305					
p (paired t-test, between)	0.276	0.623	0.107	0.134	0.332	
DBP (mmHg)						
Placebo	82 ± 10	88 ± 11	90 ± 10	87 ± 9	87 ± 9	0.502
Tolvaptan 60 mg	81 ± 8	88 ± 9	87 ± 9	85 ± 8	85 ± 8	
p (GLM between)	0.659					
p (paired t-test, between)	0.410	0.762	0.094	0.101	0.190	
Pulse rate (BPM)						
Placebo	58 ± 9	55 ± 8	55 ± 9	56 ± 9	6 0 ± 10	0.319
Tolvaptan 60 mg	58 ± 9	54 ± 9	54 ± 9	57 ± 10	60 ± 11	
p (GLM between)	0.991					
p (paired t-test, between)	0.699	0.431	0.502	0.873	0.966	

Data are given as mean ± SD. General linear model (GLM) with repeated measures was performed for comparison within and between treatment groups. Post-hoc Bonferoni test was used for comparison between L-NMMA infusion period (at the beginning/ at the end of L-NMMA infusion period) vs baseline (prior to L-NMMA infusion period) and baseline vs post infusion period (30 min/60 min after the end of L-NMMA infusion) vs baseline, none of the p- values were significant. Paired t-test was used for comparison between treatment groups

**During baseline,** no change was measured in PWV, AI, CSPB, or CDBP (Table 6).

**Table 6:** Effect of tolvaptan 60 mg at baseline, during, and after systemic inhibition of NO synthesis on pulse wave velocity (PWV), augmentation index (AI), central diastolic and systolic blood pressure (CBDP and CSBP) in a randomized, placebo-controlled, double-blind, crossover study of 18 ADPKD patients

	Prior to L-NMMA infusion (at 70 min)	During L-NMMA infusion (at 130 min)
PWV(m/s)		
Placebo	7.5 ± 1.1	8.0 ± 1.3*
Tolvaptan 60 mg	7.6 ± 1.3	8.2 ± 1.4***
p (paired t-test, between)	0.598	0.501
AI (*n* = 16)		
Placebo	21.1 ± 7.8	23.5 ± 7.3*
Tolvaptan 60 mg	21.1 ± 8.9	21.9 ± 9.1
p (paired t-test, between)	0.949	0.130
CSBP		
Placebo	132 ± 11	144 ± 18***
Tolvaptan 60 mg	128 ± 9	139 ± 16***
p (paired t-test, between)	0.084	0.124
CDBP (*n* = 17)		
Placebo	85 ± 9	93 ± 10***
Tolvaptan 60 mg	83 ± 8	88 ± 9***
p (paired t-test, between)	0.142	0.004

Data are given as mean ± SD. Paired t-test (*) was performed for comparison of means during L-NMMA infusion vs prior to L-NMMA infusion. Paired t-test was also used for comparison between tolvaptan and placebo treatment prior to L-NMMA infusion (at 70 min) and during L-NMMA infusion (at 130 min)

*p < 0.05; ***p < 0.0001

**During L-NMMA infusion,** PWV increased identically after both treatments. AI also increased, but only after placebo and to the same level as that measured after tolvaptan. Central SBP (CSBP) increased alike after both treatments. Although CDBP increased after placebo and tolvaptan treatment, CDBP remained significantly higher after placebo (*p* = 0.004).

## Discussion

The present paper reports short-term effects of tolvaptan on renal water and sodium excretion, vasoactive hormones, and brachial and central blood pressures during baseline and after systemic inhibition of the NO system in ADPKD patients.

The major results of the study were: **During baseline,** tolvaptan increased renal water excretion with no effect on sodium excretion. **During NO-inhibition**, tolvaptan decreased both water and sodium excretion to a lesser extent compared with placebo. **During the post-infusion period**, the reduced water excretion remained after tolvaptan, whereas sodium excretion returned towards baseline level. After NO-inhibition, GFR increased after placebo and remained unchanged after tolvaptan (5% vs −6%). Central diastolic BP increased to a higher level after placebo than tolvaptan. Body weight fell during tolvaptan treatment.

### ^51^ Cr-EDTA clearance

GFR fell after tolvaptan treatment in the post-infusion period 150–180 min, whereas^51^Cr-EDTA clearance was unchanged after placebo. This reduction may be attributed to changes in renal hemodynamics induced by tolvaptan [32]. Alternatively, the fall in GFR could be caused by dehydration due to increased renal water excretion after tolvaptan. However, it cannot be excluded that the elevated p-AVP level led to V1a receptor-mediated mesangial cell contraction and thus reduced GFR [33, 34]. Most likely, mild dehydration and extracellular fluid volume reduction is - at least partly - the explanation, since our patients lost approximately 1 kg in body weight after tolvaptan treatment, in contrast to an unchanged body weight during placebo. This is in good agreement with the significantly lower CDBP and the higher urinary output during tolvaptan treatment compared with placebo. The increase in p-AVP was very pronounced during tolvaptan, i. e. threefold larger than during placebo, and might have inhibited further blood pressure fall by a stimulation of the V1a receptor in the vascular bed. No increase was measured in the components in the renin-angiotensin-aldosterone system, which could have been expected after mild dehydration, but the very large increase in p-AVP seemed to have been a sufficient vasoconstrictor response during tolvaptan treatment. In our previous RCTs with tolvaptan, we did not measure a decrease in ^51^Cr-EDTA clearance [15, 16]. However, in these trials, we studied healthy control subjects, and the dosis of tolvaptan used was 15–45 mg in contrast to a dosis of 60 mg in the present study. Our results during baseline condition are in good agreement with those reported by Boertien et al. [35], where tolvaptan did not change renal hemodynamics in ADPKD with chronic kidney disease stages I-III. However, minor reduction in GFR occurred and was explained as related to acute, reversible inhibition by the tubulo-glomerular feedback mechanism [35, 36]. The TEMPO 3:4 trial and post hoc analysis of the TEMPO 3:4 trial showed that tolvaptan reduced the annual eGFR decline in ADPKD by 26% (from 3.70 to 2.72 mL/min/1.73 m^2^) [3]. Tolvaptan induced an acute decrease in eGFR during initiation of treatment. However, after discontinuation of tolvaptan, eGFR increased again. This is in good agreement with the results from the present acute study, in which GFR decreased after tolvaptan and remained lower throughout the post infusion. During NO-inhibition, we clearly demasked a reduction in ^51^Cr-EDTA clearance by tolvaptan. Thus, tolvaptan in a dose of 60 mg reduced glomerular filtration rate in ADPKD both with and without blockade of the nitric oxide synthesis.

### Tubular water excretion

As expected, tolvaptan increased renal water excretion during baseline conditions. This result is in accordance with our previous RCT in healthy subjects [15, 16]. Both studies proved increased renal water excretion following tolvaptan administration. Also, the results from the dose-response study showed that the increased renal water excretion was dose-dependent up to tolvaptan 30 mg. However, the levels of C_H2O_ and UO after tolvaptan 60 mg in ADPKD were approximately the same as after tolvaptan 30 and 45 mg in healthy subjects, which is compatible with a dysfunctional urine concentration ability in ADPKD kidneys. Thus, a higher dose of tolvaptan was necessary to induce a diuretic response in ADPKD patients. This agrees well with previous ADPKD studies [3, 21, 35, 36].

During NO inhibition, tolvaptan antagonized the antidiuretic action of L-NMMA, at least partly by an AVP-dependent mechanism. In our single-dose study, tolvaptan 15 mg promoted renal water excretion [15]. In the dose-response study, tolvaptan antagonized the antidiuretic action of L-NMMA, in part via an AVP-dependent mechanism consistent with the present study [16]. This apparent discrepancy could be explained by a difference in the administration time of the study drug, since tolvaptan was given at 6:00 AM in the single-dose study compared with 8:00 AM in the dose-response study and the present study. Thus tolvaptan antagonized the antidiuretic effect of NO-inhibition in ADPKD.

ADPKD cysts arise primarily from AVP-sensitive tubular segments and they have overexpression of V2R and AQP2 [37, 38]. Several studies have shown that tolvaptan’s diuretic action is caused by preventing AVP to bind to the V2 receptors, and thus to activate AQP2 by the second messenger cAMP. In the present study, we measured a similar level of u-AQP2 after placebo and tolvaptan, which is in agreement with our previous studies [15, 16]. This response could be explained by the 3-fold increase in p-AVP after tolvaptan. U-AQP2 reflects the action of AVP on the collecting ducts [25, 39]. Even modest changes in p-AVP have been shown to change the activity in AQP2 water channels within a few hours [40–42]. Since AQP2 is the only known apical water channel in the plasma membrane and the density of AQP2 in the plasma membrane is a rate-limiting barrier for tubular water transport [43], the compensatory and very pronounced increase in p-AVP may have counteracted tolvaptan’s effect on the V2-receptors. The very large increase in p-AVP after tolvaptan corresponds very well with our previous RCTs [15, 16].

### Tubular sodium excretion

In the present study of ADPKD, FE_Na_ decreased more after placebo than after tolvaptan during NO-inhibition. This response differs from our previous RCTs of healthy subjects [15, 16]. In both studies, FE_Na_ decreased after placebo and tolvaptan during NO-inhibition. However, in the dose-response study, FE_Na_ decreased most pronouncedly after tolvaptan [16]. Thus, in ADPKD, urinary sodium excretion seems to be lower after tolvaptan than in healthy control subjects, most likely due to an abnormal tubular function in CKD stages 1–3. In the present study, u-ENaC was the same after tolvaptan and placebo, which is in agreement with our results from a previous study in ADPKD [20]. In accordance with the present study, it showed that ADPKD patients have an abnormal tubular sodium absorption unrelated to the activity in the epithelial sodium channels [20].

### Vasoactive hormones and brachial and central blood pressure

We measured no significant changes in the components of the renin-angiotensin-aldosterone system during tolvaptan treatment, which is in agreement with our previous study in healthy subjects [15, 16]. CDBP was higher after placebo than after tolvaptan during the post-infusion period. Dehydration may have contributed to the lower level in CDBP as evidenced by body weight reduction in the tolvaptan group. These results are in good agreement with our previous studies [15, 16]. AVP has been suggested to contribute to the development of hypertension through the V2R by different ways. On one hand, AVP promotes function and expression of ENaC, and potentiates the effect of aldosterone on the collecting ducts [44, 45]. On the other hand, activation of V2R also exerts an antihypertensive effect by increasing the synthesis of NO in the collecting ducts and by increasing renal plasma flow [46–48]. However, u-ENaC was unchanged, and the NO-system was blocked in the present study. Apparently, these mechanisms were not involved in the blood pressure regulation in the present study. The very high level of AVP during tolvaptan treatment might have stimulated VR1 receptors in the vascular bed, and it cannot be excluded that this stimulation has compensated for further blood pressure decrease during tolvaptan treatment.

### Urinary excretion rate of nitrate

We found a lower baseline u-NO_3_ after tolvaptan. However, the difference was minimal and further studies with larger population are necessary to reveal any difference. During NO-inhibition u-NO_3_ decreased after both treatments confirming proper inhibition of NO-synthesis.

Tolvaptan treatment has been associated with common adverse events including polyuria, nocturia and hepatotoxicity [34]. In the present study, none of these adverse events were observed. Howerver, the observation time was relatively short and our patients had normal liver function at the inclusion. Tolvaptan dose was administered according to the ERA-EDTA recommendations, namely CKD stages 1–3 and independent of body weight [21].

## Strengths and limitations

The study design as a randomized, placebo-controlled, double-blinded, crossover is one of the major strengths of the present study. Another major strength is the use of standardized diet and fluid intake to avoid any confounding of the results. Furthermore, urinary nitrate measurement has been performed to directly document inhibition of the NO system.

The methods used to analyze u-AQP2 and u-ENaC have limitations. Emerging data suggest that the regulated fractions of these channels are excreted as urinary exosomes. These fractions were not isolated and therefore not analyzed in the present study. However, our laboratory and others have previously shown positive correlation to the function reflected by the methods used in this study.

A proportion of the patients was treated with antihypertensives during the examination period, since withdrawal of antihypertensive treatment was not ethically justified, but the treatment was the same during both placebo and tolvaptan treatment, thus a possible difference due to use of antihypertensive therapy would be expected to be minimal.

## Conclusions

During NO inhibition, tolvaptan antagonized both the antidiuretic and the antinatriuretic effect of L-NMMA, partly via an AVP-dependent mechanism. U-AQP2 was not changed by tolvaptan, presumably due to a counteracting effect of elevated p-AVP. The reduced GFR during tolvaptan most likely is caused by the reduction in extracellular fluid volume and blood pressure.

Our findings express short-term effects of tolvaptan before and during inhibition of NO. Therefore, studies with longer treatment periods are needed to clarify long-term effects of tolvaptan.

## Abbreviations

ADPKD: Autosomal Dominant Polycystic Kidney Disease
AI: Augmentation index
bBP: Brachial blood pressure
cBP: Central blood pressure
CDBP: Central diastolic blood pressure
C_H2O_: Free water clearance
CSBP: Central systolic blood pressure
DBP: Diastolic blood pressure
EMA: European Medicines Agency
FE_Na_: Fractional excretion of sodium
GFR (^51^Cr-EDTA-clearance): Glomerular filtration rate
L-NMMA: L-NG-monomethyl-arginine
NO: Nitric oxide
p-Aldo: plasma Aldosterone
p-AngII: Plasma angiotensin II
p-AVP: Plasma vasopressin
PRC: Plasma concentrations of rennin
PW: Pulse wave velocity
SBP: Systolic blood pressure
u-AQP2: Urinary aquaporin-2 channels
u-ENaCγ: Urinary epithelial sodium channels
u-NO_3_: Urinary excretion rate of nitrate
UO: Urine output
